# Current Distributions in Quantum Hall Effect Devices

**DOI:** 10.6028/jres.102.045

**Published:** 1997

**Authors:** M. E. Cage

**Affiliations:** National Institute of Standards and Technology, Gaithersburg, MD 20899-0001

**Keywords:** conducting channels, flow patterns, distributed currents, edge-channel states, quantum Hall effect, skipping orbits, two-dimensional electron gas

## Abstract

This paper addresses the question of how current is distributed within quantum Hall effect devices. Three types of flow patterns most often mentioned in the literature are considered. They are: (1) skipping orbits along the device periphery (which arise from elastic collisions off hard-walled potentials); (2) narrow conducting channels along the device sides (which are presumed to be generated from confining potentials); and (3) currents distributed throughout the device (which are assumed to arise from a combination of confining and charge-redistribution potentials). The major conclusions are that skipping orbits do not occur in quantum Hall effect devices, and that nearly all of the externally applied current is located within the device interior rather than along the device edges.

## 1. Introduction

There is considerable discussion and disagreement in the literature about how the current is distributed within quantum Hall effect devices. Some authors assume that the current is confined to skipping orbits along the device periphery, others believe it is confined to narrow edge-state channels along the device sides, and still others believe it is distributed throughout the device interior.

After a brief discussion of the quantum Hall effect, this paper investigates the nature of current patterns arising from three models that cover the above possibilities: (1) skipping orbits; (2) narrow conducting channels due to confining potentials along the device sides; and (3) distributed currents due to either a charge-redistribution potential within the device interior or to a combination of a charge-redistribution potential and confining potentials along the device sides. It is pointed out when the predictions of the three models agree or disagree with experimental results.

## 2. Integer Quantum Hall Effect

The integer quantum Hall effect [1–3] requires a fully quantized two-dimensional electron gas (2DEG). On an even integer Hall plateau, conducting electrons of the 2DEG have completely filled all the allowed spin-down and spin-up states of the lowest Landau levels, and none of the next Landau level. Negligible dissipation occurs within the interior of the 2DEG in the Hall plateau regions of high-quality devices operated at low enough currents. Within these regions the quantized Hall resistance *R*_H_ of the *i*th plateau has the value *R*_H_(*i*) = *h*/(*e*^2^*i*), where *h* is the Planck constant, *e* is the elementary charge, and *i* is an integer. In GaAs the quantum number for the first filled Landau level is *i* = 2.

## 3. Skipping Orbit Currents

**Fig. 1:**
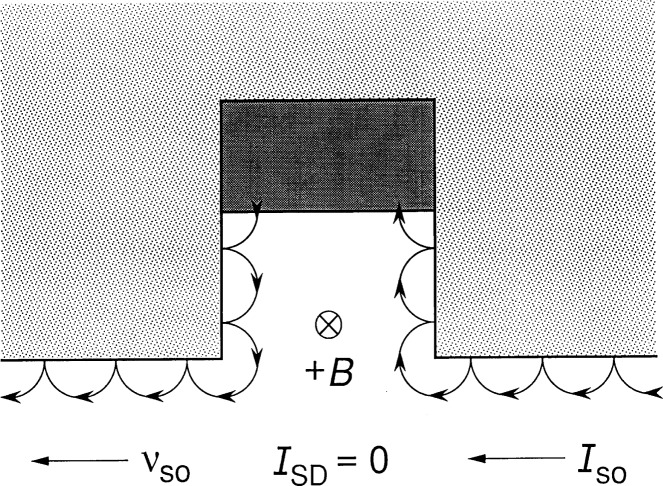
Skipping orbits of the conducting electrons around part of the device periphery when the magnetic flux density *B* points into the figure. The average velocity of the skipping orbits along the device boundary is *v*_so_, and the skipping orbit current is *I*_so_. There is no externally applied current *I*_SD_. The lightly shaded region represents a mesa etch down below the 2DEG, while the darker shaded region is an ohmic contact to the 2DEG.

Electrons of the 2DEG execute cycloidal motion when in the presence of an applied perpendicular magnetic flux density *B*. The cyclotron radius is *r*_c_ = (*ħ* / *eB*)^1/2^ for orbits of the first Landau level, in which case each electron of the 2DEG has trapped a magnetic flux quantum *h*/*e*. If: (a) the device is homogenous; (b) there is no applied current *I*_SD_ between the source S and the drain D of the device; (c) the magnetic flux density *B* is uniform; and (d) the device boundaries are represented by hard-walled confining potentials, then semiclassically the electrons are uniformly distributed throughout the device interior. The cyclotron orbital velocities vectorially tend to cancel everywhere within the device, except near the device periphery where there are skipping orbits due to elastic scattering from the hard-walled potential, as indicated in Fig. 1. An electron current therefore circulates around the device boundary in the absence of an applied current, thereby generating a measurable magnetization [4].

It is straightforward to estimate the magnitude of this skipping orbit current for a homogenous device when *I*_SD_ = 0. For a GaAs/AlGaAs heterostructure device, in which the *i* = 2 plateau occurs at a typical magnetic flux density *B* = 12.3 T, the cyclotron radius *r*_c_ = (*ħ* / *eB*)^1/2^ is 7.3 nm; the orbital angular frequency *ω*_c_ = *eB*/m* is 3.2 × 10^13^/s, where m* is the reduced mass of the electron (0.068 times the free electron mass *m*_e_); and the orbital velocity *v*_c_ = *ω*_c_*r*_c_ is 2.3 × 10^5^ m/s. The electrons travel a distance 2*r*_c_ along the device boundary in a time *t* which is one-half the cyclotron orbital period *T*_c_, so

$$t=\frac{T_{c}}{2}=\frac{1}{2f_{c}}=\frac{1}{2}\frac{2\pi }{\omega_{c}},$$ (1)

where the orbital frequency *f*_c_ is 5.1 × 10^12^ Hz and *t* is 9.8 × 10^−14^ s. The average electron velocity for skipping orbits along the device periphery is thus *v*_so_ = 2*r*_c_/*t*, or 1.5 × 10^5^ m/s. The average density of the 2DEG is *n*_s_ = *ieB*/*h*, or 5.9 × 10^15^/m^2^; and the skipping orbit current density is *J*_so_ = *n*_s_*ev*_so_, or a very large 142 A/m. The skipping orbit current is therefore

$$I_{\text{so}}\approx J_{\text{so}}r_{c}=\frac{ie^{2}B}{\pi^{2}m*},$$ (2)

which is similar to the expression *I*_so_ = *ie*^2^*B*/(2πm*) stated without proof by Thouless [5]. Thus, according to Eq. (2), 1.0 μA of skipping orbit current circulates around the device periphery for the *i* = 2 plateau at 12.3 T in the absence of an applied current.

What happens if there is an externally applied current? Consider the case where *all* the applied current executes skipping orbits. Good quantization of the *i* = 2 quantum Hall voltage *V*_H_, and small values of the longitudinal dissipative voltage *V_x_*, have been observed for applied currents as large as 200 μA [6, 7]. The above assumption would yield an enormous skipping orbit current density *J*_so_ = *I*_so_/*r*_c_ = 2.7 × 10^4^ A/m, and an average electron velocity *v*_so_ = *J*_so_/(*n*_s_*e*) = 2.9 × 10^7^ m/s, that is 9.7 % the speed of light in vacuum and 35.1 % the speed of light in GaAs. This current density and velocity is unrealistic, but it is sometimes stated [5, 8–10] that for small currents, where *V*_H_ is less than the Landau level spacing *ħω*_c_/*e*, that the applied current is an edge current, and that this edge current is concentrated within a cyclotron radius of the device boundary, and therefore undergoes skipping orbits. These authors assume that for larger currents, where *V*_H_ > *ħω*_c_/*e*, part of the applied current is a skipping orbit current and part is a bulk current within the device interior.

**Fig. 2:**
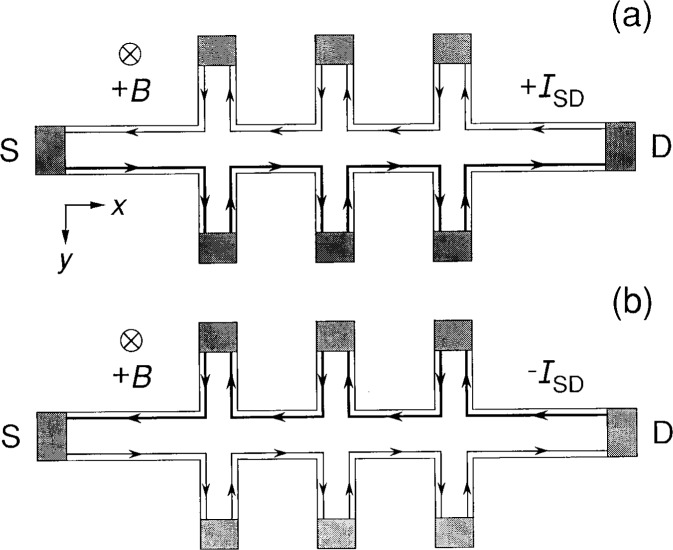
Current-carrying paths through the device if all the current were carried via skipping orbits. The thin lines are for an internally induced skipping orbit current as in Fig. 1. This current exists even when *I*_SD_ = 0. The thick lines represent internally induced current *plus* an externally applied current *I*_SD_. Figure 2(a) is for an external current of electrons entering the source contact S and exiting the drain contact D; Fig. 2(b) is for the opposite current direction. The magnetic flux density points into the figure in the positive *z* direction.

Figure 2 shows the skipping orbit flow patterns for the case of a magnetic flux density pointing into the figure, and applied currents ± *I*_SD_ small enough to satisfy the assumption that the current is contained entirely within skipping orbits. The thin lines represent an internally induced skipping orbit current, as in Fig. 1, while the thick lines are for an internally induced current *plus* the externally applied current *I*_SD_. The applied current is only along one side of the device in Fig. 2 because: (a) the Lorentz force *ev_x_B_z_* is equal and opposite to the Coulomb repulsive force *eE_y_* everywhere within the 2DEG; (b) *E_y_* = − ∇*V_y_* of the hard-walled confining potential has the opposite sign on each side of the device; and (c) only one side of the device has the appropriate sign of *E_y_* for the applied current direction.

There are two serious problems with the flow patterns of Fig. 2. The first problem is that current enters and exits the *bottom* corners of the source and drain contacts for one current direction and the *top* corners for the opposite current direction, whereas there is clear experimental evidence [11–14] that the current enters and leaves *opposite* corners of the device, and that these corners remain the *same* when the current is reversed. The second problem is that the current enters and exits every potential contact in the figure. (Even if the transmission coefficient is not unity on the first attempt to enter the contact, the reflected electrons skip along the contact and eventually enter it.) However, there is experimental evidence [15, 16] that the resistivity of the 2DEG is less than 5 × 10^−7^ Ω at 1.2 K and less than 10^−10^ Ω at 0.4 K. The latter value corresponds to a three-dimensional resistivity of less than 10^−16^ Ω cm, which is much smaller than the resistivity of any other non-superconducting material. The contacts themselves, however, are resistive in high magnetic fields. Thus, under steady-state dc conditions, the current will take the path of least resistance and *avoid* the potential contacts. (This assumes that no current is drawn through the potential probe contacts by an external measurement system, and that the time is long enough after current reversal for the device potentials to have reached equilibrium.)

Skipping orbits do not seem feasible in the quantum Hall effect for the two reasons just given, and for reasons that will be presented in Secs. 4.3 and 5.3.

## 4. Narrow Conducting Channel Currents

Even if skipping orbits do not occur in the quantum Hall effect, current will still circulate around the device periphery because a confining potential must exist to prevent electrons of the 2DEG from spilling out of mesa-etched devices when the etching extends below the depth of the 2DEG. We will see in this section that confining potentials along the sides of the device create narrow channels, along which, current can flow.

### 4.1 Eigenstates of the 2DEG

**Fig. 3:**
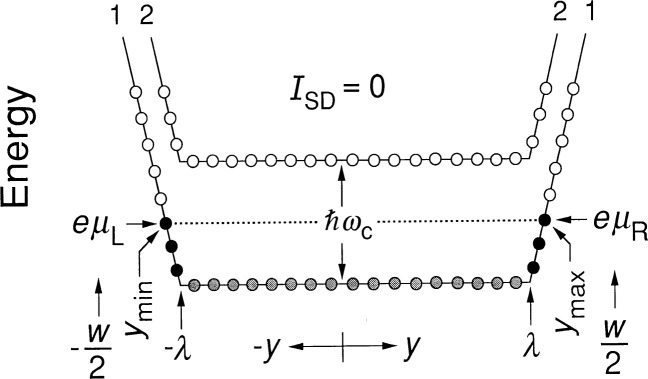
Schematic drawing of the energies of allowed eigenstates across the device width *w* for a linearly shaped confining potential of greatly exaggerated spatial extent *λ* and no applied current *I*_SD_. Only a few eigenstates are indicated. The *x*-axis points along the center line of the device. The electrochemical potential *μ* is the same on both sides of the device for this case. Eigenstates of the lowest Landau level 1 are filled between *y*_max_ = − *y*_min_. Shaded circles are for occupied states that do not contribute to the current, black circles are current-carrying occupied states, and open circles are unoccupied states.

Reference [17] explains how the confining potential arises from electron surface charges on the side of the mesa and a charge-depletion region near the device edge. Note that this potential is *negative*. Therefore we assume a negative confining potential *V*_c_ of finite spatial extent *λ* on each side of the device. The energy of an electron in this confining potential is then ***ℇ***_c_ = *qV*_c_ = − *eV*_c_. Figure 3 is a schematic drawing of the electron energies, plotted as a function of *y*, across the device width *w* for a confining potential of greatly exaggerated spatial extent *λ*. (For convenience, the figure shows a linearly-shaped confining potential, but we will use a more realistically shaped parabolic confining potential in the calculations.) No current exists within the device interior in Fig. 3 because the potential gradient is zero between −*λ* and *λ*.

The electrons in the 2DEG occupy unique quantum eigenstates, indicated as circles in Fig. 3. The usual method of defining these states is to represent their wavefunctions in the Landau gauge as normalized products of Hermite polynomials across the device multiplied by plane waves propagating down a length *L_x_* of the device [18–20]. Let us consider only even-*i* quantum Hall plateaus. The energy eigenvalue ***ℇ****_N_* of each state in Landau level *N* is then

$${ℰ}_{N}\left(y_{0}\right)=\left(N+\frac{1}{2}\right)\hbar \omega_{c}+ey_{0}E\left(y_{0}\right)+\frac{1}{2}m*v_{x}^{2}\left(y_{0}\right),$$ (3)

where

$$y_{0}=\left(v_{x}/\omega_{c}+\ell_{B}^{2}k_{x}\right)$$ (4)

is the center-of-mass position of each state undergoing cycloidal motion, − *w*/2 < *y*_0_ < *w*/2; *v_x_*(*y*) = *E*(*y*)/*B* is the electron drift velocity down the device; *ℓ_B_* = (*ħ* / *eB*)^1/2^ is the magnetic length, and is equal to the cyclotron radius *r*_c_ for the first Landau level; and *k_x_* = 2π*N_k_*/*L_x_* is the wavevector for the state located at position *y*_0_ with an associated positive or negative integer quantum number *N_k_*. The eigenstates are represented by the quantum numbers (*N*,*N_k_*), and the wave-function for each state is

$$\begin{array}{l} \psi_{N,N_{k}}\left(x,y\right)=\frac{1}{{\left(L_{x}\right)}^{1/2}}e^{i2\pi N_{k}x/L_{x}}\frac{1}{{\left(2^{N}N!\right)}^{1/2}}\\ \times \frac{1}{{\left(\pi \ell_{B}^{2}\right)}^{1/4}}e^{-{\left(y-y_{0}\right)}^{2}/2\ell_{B}^{2}}H_{N}\left[\left(y-y_{0}\right)/\ell_{B}\right], \end{array}$$ (5)

where *H_N_*[(*y* − *y*_0_)/*ℓ_B_*] is a Hermite polynomial.

The eigenstates, represented as circles located at cyclotron center-of-mass positions *y*_0_, are shown in Fig. 3 for the first (*N* = 0), (*i* = 2) Landau level and the second (*N* = 1), (*i* = 4) Landau level. Only a few of the eigenstates are indicated. The spatial extent of each eigenstate [18–20] is $\pm \;\ell_{B}\sqrt{2N+1}$, which is equal to 2*r*_c_ for the first Landau level; the spatial separation between adjacent states in a constant electric field is $\Delta y_{0}=2\pi \ell_{B}^{2}/L_{x}$ from Eq. (4); and the energy separation between adjacent Landau levels is *ħω*_c_.

The magnetic flux density has been adjusted in Fig. 3 so that all the allowed states of the first Landau level are filled and none of the second. The Landau level is midway across a mobility gap [21]. Only localized states due to imperfections and impurities are being filled. These localized states (not shown in the figure) do not affect the quantum Hall voltage because the states are stationary [22–23] (non-conducting). Since there is no applied current, and therefore no Hall voltage, the Fermi energy ***ℇ***_F_ is constant across the device width, and is located halfway between Landau levels, as indicated by the dotted line in Fig. 3. Under these conditions, states of the lowest Landau level are occupied up to the Fermi energy ***ℇ***_F_ = *ħω*_c_, and no states are occupied in the second Landau level. The occupied states are located between *y*_max_, and *y*_min_, and in this case |*y*_max_| = |*y*_min_|. Shaded circles are for occupied states that do not contribute to the current because *E_y_* is zero; black circles represent current-carrying occupied states where *E_y_* ≠ 0; and open circles are unoccupied states.

Voltage probes located along the sides of the device measure the electrochemical potential *μ* of the 2DEG [24]. We assume that the probe potential is that of the nearest occupied conducting state on that side of the device, i.e., the state at *y*_max_ or *y*_min_. We also assume that the conducting states at *y*_max_ or *y*_min_ are far enough from the mesa-etched sides that the shape of the confining potential is not significantly altered by the boundary conditions, and that the potential and the electric field are continuous across the mesa-etched interface. In the special case of Fig. 3 where *I*_SD_ = 0, *eμ*_R_ = *eμ*_L_ = ***ℇ***_F_ on either side of the device.

### 4.2 Confining Potential

A current circulates around the device periphery within the narrow regions containing the black-circle eigenstates of Fig. 3 when there is an external magnetic field. The existence of this current is predicted by edge-channel models [8, 24–31], and is verified by experiment [32–37]. (Edge *channel* is a better description of this phenomenon than edge *state* because each conducting channel is composed of many eigenstates.) Most edge-channel models recognize that the confining potentials have a finite extent, but then assume that the confining potentials are hard-walled at ± *w*/2 when making calculations, and that *y*_max_ = *w*/2 − *r*_c_ = − *y*_min_.

We consider the more realistic case where the confining potentials have a finite spatial extent, rather than hard walls. For simplicity, the confining potentials shown in the schematic drawing of Fig. 3 are linear, but we will assume parabolically-shaped confining potentials in the calculations. These parabolically-shaped confining potentials arise from homogeneous charge-depletion regions of spatial extent *Δ*. The confining potentials of Fig. 3 have origins at *y* = *λ* = *w*/2 − *Δ* and *y* = − *λ* = − *w*/2 + *Δ*. The value of *Δ* has been determined in an experiment by Choi, Tsui, and Alavi [38]. They used one-dimensional localization theory to evaluate conduction in narrowly-constricted channels of GaAs/AlGaAs heterostructures at very small magnetic flux densities, and found that *Δ* = 0.5 μm ± 0.2 μm. (This result is consistent with observations that devices having widths less than 0.5 μm cease to conduct altogether at temperatures below 4.2 K [38], even at high magnetic flux densities [39] where the 2DEG penetrates into the depletion region.) We choose the value of the charge-depletion depth to be their average value 0.5 μm. The remaining parameter to define the confining potential is *V*_m_, the value of the confining potential at ± *w*/2. The confining potential exists even at zero applied current and zero magnetic flux density. Also, there are many impurity states between the valence and conduction bands. A reasonable value of *V*_m_ is one-half of the separation voltage between the valence and conduction bands, which is *V*_m_ = 0.75 V in GaAs at 1 K [40]. This value is comparable to the 0.8 V used by Choi, Tsui, and Alavi [38].

The equations for the parabolic confining potential *V*_c_ and its electric field *E*_c_ = − ∇*V*_c_ are therefore

$$\begin{array}{c} V_{c}\left(y\right)=-a{\left(y-\lambda \right)}^{2}\text{and}\;E_{c}\left(y\right)=2a\left(y-\lambda \right)\\ \text{for}\;\lambda \leq y\leq \frac{w}{2}, \end{array}$$ (6a)

$$\begin{array}{c} V_{c}\left(y\right)=0\;\text{and}\;E_{c}\left(y\right)=0\\ \text{for}\;-\lambda <y<\lambda , \end{array}$$ (6b)

$$\begin{array}{c} V_{c}\left(y\right)=-a{\left(y+\lambda \right)}^{2}\;\text{and}\;E_{c}\left(y\right)=2a\left(y+\lambda \right)\\ \text{for}\;-\frac{w}{2}\leq -y\leq -\lambda , \end{array}$$ (6c)

where *a* = *V*_m_/*Δ*^2^ = 3.0 × 10^12^ V/m^2^ for *Δ* = 0.5 μm and *V*_m_ = 0.75 V, and

$$\lambda =\frac{w}{2}-\Delta .$$ (7)

### 4.3 Confining Potential at *I*_SD_ = 0 μA

Given the above equations and the values of *Δ* and *V*_m_, much information can be deduced about the current distribution when *I*_SD_ = 0 μA and the magnetic flux density is adjusted to be halfway between Landau levels and on the *i* = 2 quantum Hall plateau, as is the case in Fig. 3. Under these conditions

$$\begin{array}{c} {ℰ}_{c}\left(y_{\max}\right)=\frac{\hbar \omega_{c}}{2}=-eV_{c}\left(y_{\max}\right)=ea{\left(y_{\max}-\lambda \right)}^{2}\\ =e\frac{V_{m}}{\Delta^{2}}{\left(y_{\max}-\frac{w}{2}+\Delta \right)}^{2}, \end{array}$$ (8)

if we measure the energy ***ℇ***_c_(*y*_max_) relative to ***ℇ***_c_(0). Thus

$$y_{\max}=-y_{\min}=199.559\;\mu \text{m}$$ (9)

and

$$\frac{w}{2}-y_{\max}=0.441\;\mu \text{m}$$ (10)

for a 400 μm wide quantized Hall resistance standard device at a magnetic flux density of 12.3 T. The electric field *E*_c_(*y*) from Eq. (6a) is 3.6 × 10^5^ V/m at *y*_max_ = 199.559 μm. The current density along the *x*-axis at position *y* is *J*_c_(*y*) = *σ_xx_E_x_* (*y*) + *σ_xy_E_c_* (*y*) = *σ_xy_E_c_* (*y*) in the absence of significant dissipative scattering [41], where the off-diagonal conductivity tensor component is *σ_xy_* = *ie*^2^/*h* = 1/*R*_H_ = 1/12 906.4 Ω for the *i* = 2 plateau. *J*_c_(*y*_max_) = 27.5 A/m for this case.

The total current carried by the occupied states of the right-hand side (rhs) confining potential is

$$\begin{array}{l} I_{c}\left(\text{rhs}\right)=\int_{\lambda }^{y_{\max}}J_{c}\left(y\right)dy=\int_{\lambda }^{y_{\max}}\sigma_{xy}E_{c}\left(y\right)dy=\\ -\frac{1}{R_{H}}\left[V_{c}\left(y_{\max}\right)-V_{c}\left(\lambda \right)\right]=-\frac{V_{c}\left(y_{\max}\right)}{R_{H}}, \end{array}$$ (11)

where *V*_c_(*y*_max_) = − *a*(*y*_max_ − *λ*)^2^. Similarly for the left-hand side (lhs),

$$\begin{array}{l} I_{c}\left(\text{lhs}\right)=\int_{y_{\min}}^{-\lambda }J_{c}\left(y\right)dy=\int_{y_{\min}}^{-\lambda }\sigma_{xy}E_{c}\left(y\right)dy=\\ -\frac{1}{R_{H}}\left[V_{c}\left(-\lambda \right)-V_{c}\left(y_{\min}\right)\right]=\frac{V_{c}\left(y_{\min}\right)}{R_{H}}, \end{array}$$ (12)

where *V*_c_(*y*_min_) = − *a*(*y*_min_ + *λ*)^2^. It follows from Eqs. (8), (11), and (12) that

$$I_{c}\left(\text{rhs}\right)=\frac{\hbar \omega_{c}}{2eR_{H}}=\frac{ie^{2}B}{4\pi m*}=0.81\;\mu \text{A}\;=-I_{c}\left(\text{lhs}\right)$$ (13)

for the 12 906.4 Ω, *i* = 2 plateau at 12.3 T and *I*_SD_ = 0 μA. This rather large 0.8 μA edge-channel current circulating around the device is comparable to the 1.0 μA current obtained in Sec. 3 for skipping orbits. Note from Eq. (13) that the current is independent of the device width *w* and of the confining potential parameters *Δ* and *V*_m_ if *w* > 2*Δ* and *I*_SD_ = 0 μA, whereas *y*_max_ and *y*_min_ depends on *w*, *Δ*, *V*_m_, and *I*_SD_.

We see from Eq. (10) that the maximum spatial extent of the current-carrying states is *60 times* farther away from the sides of the device than predicted for the 7.3 nm radius skipping orbits; thus there is no need to invoke skipping orbits. Indeed, if there *were* skipping orbits, the electric field *E*_c_(*y*) calculated from Eq. (6a) would be an enormous 2.96 × 10^6^ V/m at *y*_max_ = *w*/2 − *r*_c_. Also, the current density *J*_c_(*w*/2 − *r*_c_) would be a very large 229 A/m, even for this case with no applied current. As a final argument against skipping orbits, note in Fig. 3 that there are unoccupied Landau eigenstates (and localized states not shown in the figure) at *y*_max_ and *y*_min_. If the states at *y*_max_ and *y*_min_ executed skipping orbits, then scattering into unoccupied states could occur at every reflection, and this scattering need not be elastic. Hence dissipation could occur. The electron current paths will surely adjust to minimize this dissipation.

**Fig. 4:**
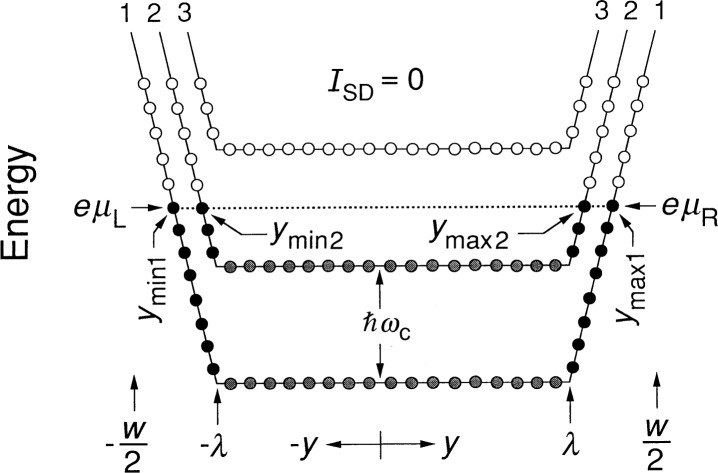
Similar to Fig. 3, but two Landau levels are filled and there are now two values of *y*_max_ and two values of *y*_min_ = − *y*_max_.

Next, consider the case in Fig. 4 where *I*_SD_ = 0 μA and two Landau levels are occupied, i.e., the case for the *i* = 4 plateau. The energies of the highest-filled eigenstates of the first and second Landau levels are (3/2)*ħω*_c_ and (1/2)*ħω*_c_, respectively. We can use Eq. (8) to calculate *y*_max1_ = − *y*_min1_ and *y*_max2_ = − *y*_min2_ for a typical 5.5 T magnetic flux density on the *i* = 4 plateau. The values are 199.568 μm and 199.540 μm. The electric fields and current densities at the two values of *y*_max_ are 4.1 × 10^5^ V/m and 63.6 A/m, and 2.4 × 10^5^ V/m and 36.7 A/m, respectively. Three-fourths of the 1.45 μA current circulating around the device periphery with no applied current is due to electrons in the lowest Landau level.

We can also estimate the spatial separation of the two conducting edge-channels for the first two Landau levels: they are separated at the Fermi level energy by *y*_max1_ − *y*_max2_, and the total spatial extents of their wave-functions are approximately $\ell_{B}\left(\sqrt{2N+1}+\sqrt{2N^{\prime }+1}\right)$. Their separations for this example (where *N* = 0, *N′* = 1, and *B* = 5.5 T) are 8 % farther apart than the spatial extents; thus we predict that the edge-channels are physically separated from each other. Buttiker [28] used a different approach in his Eq. (56) to arrive at a similar result.

### 4.4 Confining Potential at *I*_SD_ ≠ 0 μA

**Fig. 5:**
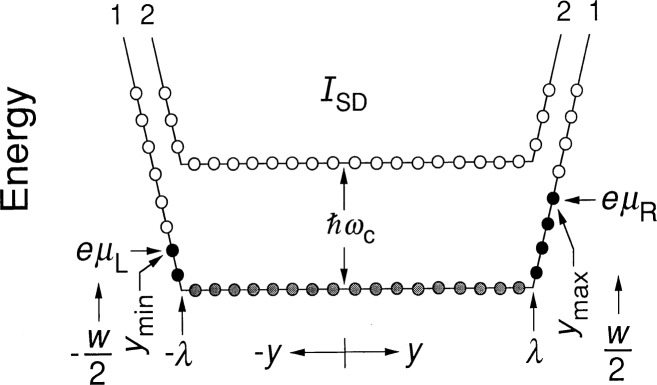
Similar to Fig. 3, but with a small value of applied current *I*_SD_. The chemical potential is now different on the two sides of the device.

Figure 5 shows the situation for an applied current *I*_SD_, assuming all the current is within the confining potential regions *λ* to *y*_max_ and − *λ* to *y*_min_. (This is a reasonable assumption for very small applied currents in the picoampere and nanoampere ranges.) The value of *y*_max_ increases with increasing *I*_SD_, and additional eigenstates are occupied on the right-hand side of the device. Fewer states are occupied on the left side, and <*y*_min_< decreases. The chemical potentials are now different on the two sides of the device, and the Hall voltage is *V*_H_ = *R*_H_*I*_SD_ = (*μ*_L_ − *μ*_R_). If the confining potentials were linear we could determine the values of *y*_max_ and *y*_min_ for any small applied current because one-half the Hall voltage would appear on each side of the device. However, we require more information for other potential shapes because a range of *y*_max_ values exists for which corresponding values of *y*_min_ can be obtained that also provides the correct quantum Hall voltage.

**Fig. 6:**
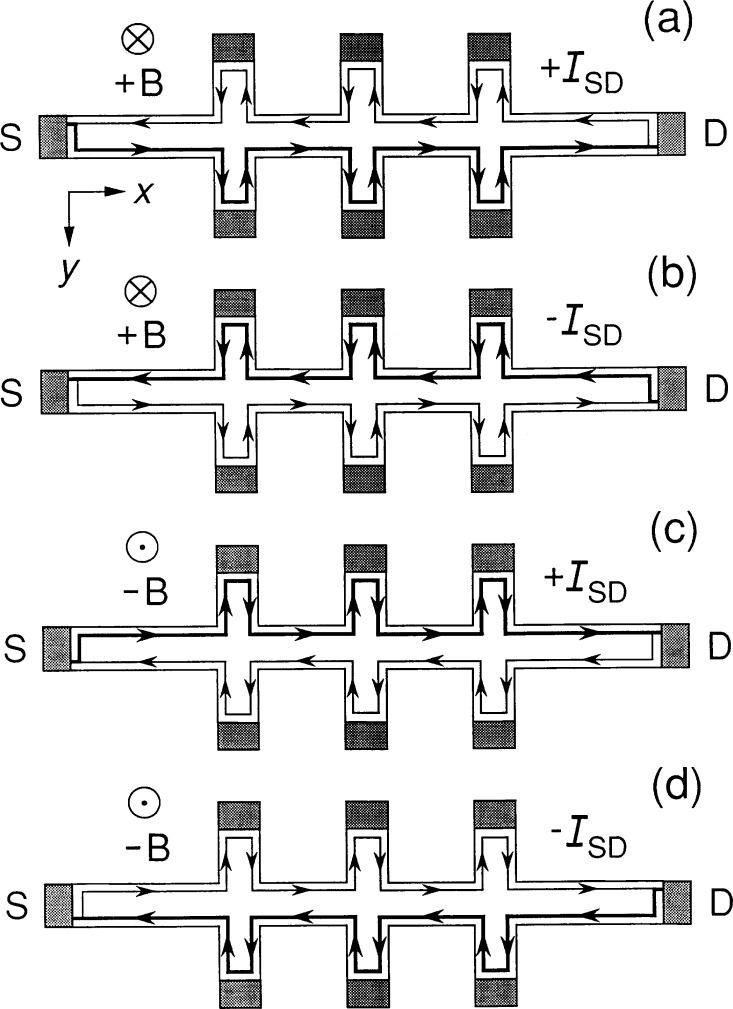
Current-carrying paths through the device if all the current is via edge-channels generated by the confining potentials, as in Fig. 5. The thick lines represent the side of the device where more of the current flows. There is less current through the thin lines. The magnetic flux density points into the device in the positive *z* direction in Figs. (6a) and (6b), and out of the device in Figs. (6c) and (6d).

Figure 6 is a schematic of the current-carrying paths through the device if all the current is carried via edge-channels generated by confining potentials, as is the case in Fig. 5. The paths are for ± *I*_SD_ and ± *B*. Current circulates around the device, and it would be equal and opposite on either side of the device if *I*_SD_ = 0 μA, as in Fig. 3. More of the current is carried by the thick-line paths when there is an applied current. The applied current *I*_SD_ enters and exits opposite corners of the device, in agreement with experiment [11–14]. These opposite corners are interchanged on magnetic field reversal. There are no skipping orbits in Fig. 6 because *y*_max_ < (*w*/2 − *r*_c_).

I suggest in Fig. 6 that the dc current takes the path of least resistance and avoids the potential contacts once the device reaches steady-state conditions because the resistivity of the 2DEG is so much smaller than the resistivity of the potential contacts [15, 16]. Perhaps, however, there is a physical requirement for the current to enter the potential contacts, as assumed in the Landauer-Buttiker formalism [26–28] with transmission and reflection coefficients at the contacts.

We assume in Fig. 5 that *V*_H_ << *ħω*_c_/*e* and *I*_SD_ << *ħω*_c_/*eR*_H_ since there is no significant electric field or current in the interior between − *λ* and *λ*. For the *i* = 2 plateau at 12.3 T this means that *I*_SD_ << 1.6 μA. This condition is easily satisfied in edge-channel experiments [32–37] where the current is typically less than 50 nA. This condition is also consistent with the experiment of Kane, Tsui, and Weimann [42] where they observed a change in the behavior of the longitudinal resistance *R_x_* between filamentary and bulk-like currents at 1.5 μA: at 1.5 μA, *V*_H_ = *ħω*_c_/*e* in their device, and *R_x_* then scaled inversely with the device width *w*, as predicted from the bulk current condition *R_x_* = *ρ_xx_L_x_*/*w*, where *ρ_xx_* is the resistivity and *L_x_* is the length down the device between *V_x_* probes.

This section has dealt with zero or very small applied currents, such that *eV*_H_ << *ħω*_c_. That is often not the case. Indeed, we have used applied currents as large as 200 μA and still observed reasonably good quantization of the Hall voltage *V*_H_ and small values of the longitudinal dissipative voltage *V_x_* = *R_x_I*_SD_ [6, 7]. A 200 μA current for the *i* = 2 plateau yields *eV*_H_ = 123 *ħω*_c_, which means that the assumption of Fig. 5 that current does not exist in the device interior no longer holds.

## 5. Distributed Currents

There is experimental evidence [13, 42–46] that significant current exists within the device interior when *eV*_H_ > *ħω*_c_. This is often referred to as a bulk current. Some of the experiments [43–46] used contacts within the device interior, leading to concern that the contacts perturbed the current distribution, and speculation that the apparent bulk current was really due to edge-channels at each internal contact. However, part of the experiment of Kane, Tsui, and Weimann [42] used no internal contacts, and the experiment of Fontein et al. [13] was a contactless measurement. Those two experiments clearly indicate the existence of internal currents. This section will therefore consider the case of current within the device interior.

### 5.1 Charge-Redistribution Potential

Initially we will ignore the confining potentials, but they will then be included in Sec 5.2. Several theories have been used for bulk currents, such as a classical electrodynamics model using local conductivity tensors [47, 48], and percolation down the device along paths of constant potential [49–52]. A model will be used here in which the applied current *I*_SD_ induces a potential distribution within the device that extends across most of the device width *w*.

The Lorentz force exerted on the conducting electrons of the 2DEG causes an increase in the density of electrons on one side of the device and a decrease on the other side. Thus, there are deviations, − *eδσ* (*y*), from the average surface charge density − *en*_s_ = − *ie*^2^*B*/*h* of the 2DEG across the device width. The charge-redistribution − *eδσ* (*y*) can be represented as a sequence of line charges, where the sequence is across the device in the ± *y* directions and the line charges point along the device in the ± *x* directions. A logarithmic charge-redistribution potential *V*_r_(*y*) across the device results from this sequence of line charges.

MacDonald, Rice, and Brinkman [53] expressed *V*_r_(*y*) self-consistently in terms of the charge-redistribution. Riess [54] extended this potential to a 2DEG with finite thickness. Thouless [55] found an analytic logarithmic approximation of *V*_r_(*y*) far from the sides of the device. The charge-redistribution potential is infinite at the physical device edge, so Beenakker and van Houten [29] approximated the near-edge behavior by introducing a cut-off near the device side and a linear extrapolation to the edge. Balaban, Meirav, and Shtrikman [56] used a quadratic extrapolation near the device sides, and a cut-off that was the same at both edges of the device and at all currents. Their cut-off distance from the device edge was the magnetic length *ℓ_B_* = (*ħ* / *eB*)^1/2^ (which is the cyclotron radius *r*_c_ for skipping orbits of electrons in the first Landau orbit). Cage and Lavine [17] used the same form for the potential as Balaban et al. [56], but a different geometrical factor and the very different cut-off values *δ*_max_ = *w*/2 − *y*_max_ and *δ*_min_ = *w*/2 + *y*_min_ (which differ on either side of the device, vary with applied current, and depend on the magnetic flux density direction). No extrapolation to the device edges was used; the occupied Landau eigenstates were assumed to be far enough from the device edges to be unaffected by the conditions that the potential and electric fields are continuous across the boundary at the mesa edge.

The charge-redistribution potential of Cage and Lavine [17] is

$$V_{r}\left(y\right)=-\frac{I_{r}R_{H}}{2}{\left[\ln\frac{y_{\max}+w/2}{w/2-y_{\max}}\right]}^{-1}\ln\left|\frac{y+w/2}{y-w/2}\right|,$$ (14)

for

$$-\frac{w}{2}<y_{\min}\leq y\leq y_{\max}<\frac{w}{2}$$

where

$$I_{r}=I_{\text{SD}}-I_{c}\left(\text{rhs}\right)-I_{c}\left(\text{lhs}\right).$$ (15)

*I*_c_(rhs) and *I*_c_(lhs) are defined by Eqs. (11) and (12), and are zero if there is no confining potential. The geometry factor *G* is

$$G\left(w,\;y_{\max}\right)={\left[\ln\frac{y_{\max}+w/2}{w/2-y_{\max}}\right]}^{-1}.$$ (16)

The charge-redistribution electric field *E*_r_ = − ∇*V*_r_ is

$$E_{r}\left(y\right)=\frac{I_{r}R_{H}}{2}G\frac{w}{\left[{\left(w/2\right)}^{2}-y^{2}\right]}.$$ (17)

**Fig. 7:**
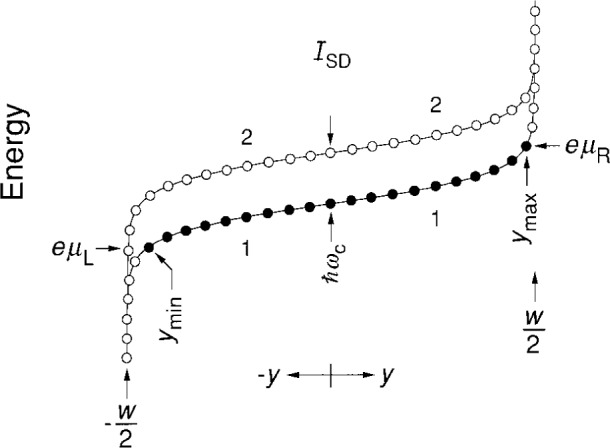
Schematic drawing of the energies of allowed eigenstates of two Landau levels for the applied current-induced charge-redistribution potential defined by Eqs. (14) and (15). Only a few eigenstates are indicated. Eigenstates of the lowest Landau level are filled between *y*_min_ and *y*_max_. Black circles are current-carrying occupied states that contribute to the chemical potentials *μ*_R_ and *μ*_L_ on either side of the device. Open circles are unoccupied states.

Figure 7 is a schematic drawing of the energy ***ℇ***_r_ = *qV*_r_ = − *eV*_r_ across the device width *w* for two Landau levels when using the charge-redistribution potential defined by Eqs. (14) and (15). A few energy eigenstates are also shown. *V*_r_(*y*) is infinite at ± *w*/2, but that does not matter because there are no occupied eigenstates beyond *y*_max_ and *y*_min_. Only the occupied con ducting states of the first Landau level between *y*_max_ and *y*_min_ (indicated in black) contribute to the chemical potentials on either side of the device and to the quantum Hall voltage. The potential is therefore finite and well-behaved in the region of interest.

This defines a realistic charge-redistribution potential. However, just as in Sec. 4.4 for the confining potential, additional information is required to uniquely determine the values of *y*_max_ and *y*_min_ for any applied current *I*_SD_ because a range of *y*_max_ values exists for which corresponding values of *y*_min_ can be obtained that provide the correct quantum Hall voltage.

### 5.2 Charge-Redistribution Potential and Confining Potentials

What if the current exists along the device edges *and* within the interior? Many authors have considered this possibility [42, 57–65]. The case considered here involves a confining potential *V*_c_(*y*) defined by Eqs. (6) and (7) and located on either side of the device, and an applied current-dependent charge-redistribution potential *V*_r_(*y*) defined by Eqs. (14), (15), (11), and (12). Note from Eqs. (15), (11), and (12) that *V*_r_(*y*) is zero everywhere if there is no applied current *I*_SD_ because *I*_c_(rhs) = − *I*_c_(lhs) for that situation.

**Fig. 8:**
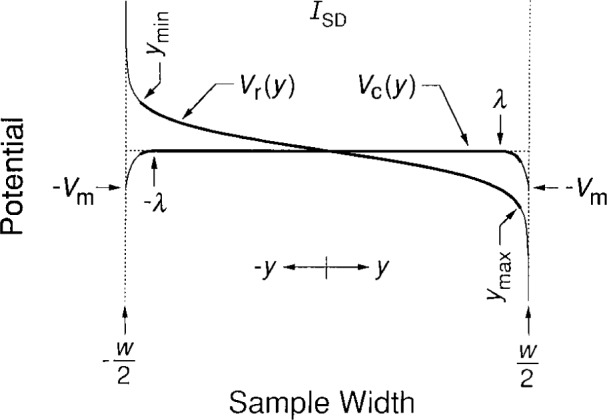
Schematic drawing of the confining potentials *V*_c_(*y*) and the charge-redistribution potential *V*_r_(*y*). The thin lines are the two types of potentials. Thick lines indicate the regions of occupied eigenstates, which extend between *y*_max_ and *y*_min_. The origins of the confining potentials at ± *λ* are greatly exaggerated for clarity, and *y*_max_ and *y*_min_ are much farther from the device sides than in an actual example.

Figure 8 is a schematic diagram of these confining and charge-redistribution potentials. The thick lines indicate the regions of occupied eigenstates, which extend between *y*_max_ and *y*_min_. The values of *y*_max_ and *y*_min_ are the same for the confining and charge-redistribution potentials. Increasing the current shifts the thick lines closer to one side of the device and farther away from the other side. The sign of the shift depends on the magnetic field and applied current directions.

The electrical transport properties depend on the *total* potential *V*_t_(*y*), but if we can unambiguously separate *V*_t_(*y*) into the confining and charge-redistribution potential components then

$$V_{t}\left(y\right)=V_{c}\left(y\right)+V_{r}\left(y\right).$$ (18)

Most of the information required for Eq. (18) is known. The potentials *V*_c_(*y*) and *V*_r_(*y*) are defined by Eqs. (6), (7), (11), (12), (14), and (15). For a given device we know the applied current *I*_SD_ and the device width *w*. The current-independent parameters for the confining potential are *Δ* = 0.5 μm and *V*_m_ = 0.75 V. If the geometry factor *G* of the charge-redistribution potential is assumed to be current-independent, then evaluation of Eq. (16) with *y*_max_ = 199.559 μm and *w* = 400 μm, found for the case when *I*_SD_ = 0 μA in Sec. 4.3, gives the value *G* = 0.147.

The problem remains, however, that just as in the cases of separate confining potentials and charge-redistribution potentials, there are still two free parameters *y*_max_ and *y*_min_. Ordinarily, it is impossible to uniquely determine the values of *y*_max_ and *y*_min_ since the only other information is that the Hall voltage *V*_H_ is

$$V_{H}=R_{H}I_{\text{SD}}=V_{t}\left(y_{\min}\right)-V_{t}\left(y_{\min}\right),$$ (19)

and there is a range of values for *y*_max_ and *y*_min_ that satisfy this equation. It *was* possible, however, in an experiment of Cage and Lavine [20], to determine *y*_max_, and to thereby obtain a unique value of *y*_min_ from Eq. (19).

Cage and Lavine [20] measured the quantized longitudinal voltage drops *V_x_* along a GaAs/AlGaAs device on the *i* = 2 plateau at 12.3 T for high currents in the breakdown regime [6, 7, 19, 66–70], and deduced the maximum electric field *E*_max_ from a quasi-elastic inter-Landau level scattering model [18–20, 71]. The result was

$$E_{\max}=1.1\times 10^{6}\;V/m\;\text{at}\;I_{\text{SD}}=215\;\mu \text{A}$$ (20)

to excite the lowest *V_x_* quantum voltage.

Since *E*(*y*) = − ∇*V*(*y*), it follows from Eq. (18) that

$$E_{t}\left(y_{\max}\right)=E_{c}\left(y_{\max}\right)+E_{r}\left(y_{\max}\right).$$ (21)

It is clear from Fig. 8 that *E*_max_ will occur at the side of the device where *V*_c_(*y*) and *V*_r_(*y*) combine to yield the largest value. This is at *y*_max_; hence

$$E_{t}\left(y_{\max}\right)=E_{\max}.$$ (22)

We can therefore use the electric field Eqs. (6), (17), (21), and (22) to determine *y*_max_, and then the potential Eqs. (6), (7), (14), and (15), plus the quantum Hall voltage Eq. (19), to obtain *y*_min_ for the device of Ref. [20]. (Note that changing the values of *y*_max_ and *y*_min_ also alters the values of *I*_c_(rhs) and *I*_c_(lhs), and thereby the value of *I*_r_.) There are now no free parameters, and one can obtain unique solutions to the total potential *V*_t_(*y*) and to other transport properties.

### 5.3 Results for the Total Potential

The calculations for a 400 μm wide device on the *i* = 2 plateau at 12.3 T were done in Ref. [17]. The values of *y*_max_ and *y*_min_ at 215 μA were 199.599 μm and − 199.515 μm, respectively, which are 55 and 66 cyclotron radii away from the device edges. Therefore, there are *no* skipping orbits. Note from Sec. 4.3 that *y*_max_ and *y*_min_ were both 60 cyclotron radii away from the edges when *I*_SD_ = 0 μA; thus *y*_max_ increased by only 40 nm between *I*_SD_ = 0 μA and 215 μA.

**Fig. 9:**
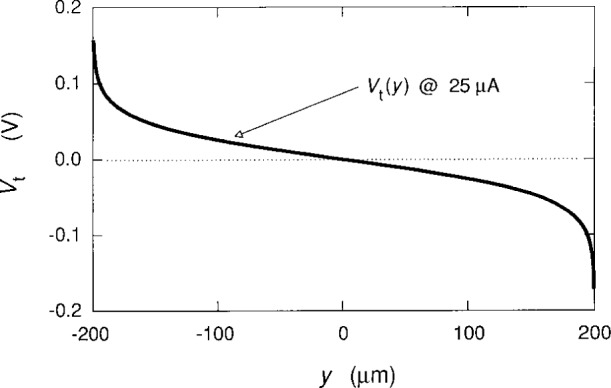
The total potential *V*_r_(*y*) at *I*_SD_ = 25 μA for the *i* = 2 plateau. The conducting states of the potential extend between *y*_min_ = − 199.554 μm and *y*_max_ = 199.564 μm. The quantum Hall voltage is 0.323 V.

Cage and Lavine [17] also did the calculations for *I*_SD_ = 25 μA, which is a typical current in quantized Hall resistance standards measurements. They used a linear interpolation of *y*_max_ between the values for *I*_SD_ = 0 μA. For convenience, their plot of the total potential *V*_t_(*y*) is reproduced in Fig. 9. This predicted potential is in excellent agreement with the contactless experimental measurements shown in Fig. 6 of Fontein et al. [13], which verifies that this model of a total potential composed of confining potentials plus a charge-redistribution potential is reasonable.

The model of Cage and Lavine [17] appeared to not conserve charge. Slightly more electrons were redistributed towards the + *y* side of the device than were removed from the − *y* side. Therefore, there seemed to be an unaccounted excess of electrons. They pointed out that charge conservation could be accomplished by adjusting the origin to the right until the total potential *V*_t_(*y*) and the charge-redistribution function

$$-e\delta \sigma \left(y\right)=-e\frac{im*}{hB}\frac{d^{2}}{dy^{2}}V_{t}\left(y\right)$$ (23)

were self-consistent. They did not add this complication, however, since the potential distribution was already in good agreement with the experiment of Fontein et al. [13]. The charge is actually conserved in their model without adjustment of the origin because eigenstates of the Landau levels in the 2DEG become occupied/unoccupied by electrons on each side of the device near *y*_max_ and *y*_min_ rapidly tunneling from/to the ionized donor atoms in the AlGaAs layer located above the 2DEG and maintaining charge equilibrium, even at low temperatures [72]; thus the net charge within the GaAs and AlGaAs layers is always zero.

Once *V*_t_(*y*) = *V*_c_(*y*) + *V*_r_(*y*) is known, the electric fields *E*_c_(*y*) = − ∇*V*_c_(*y*) and *E*_r_(*y*) = − ∇*V*_r_(*y*) can be determined from Eqs. (6) and (17). The current density *J*_t_(*y*) for electrons moving in the positive *x* direction is then

$$J_{t}\left(y\right)=\sigma_{xy}E_{t}\left(y\right)=\frac{ie^{2}}{h}\left[E_{c}\left(y\right)+E_{r}\left(y\right)\right],$$ (24)

and the current *I*(*y*) is

$$I\left(y\right)=\int_{0}^{y}J_{t}\left(y\right)\;dy=-\frac{V_{t}\left(y\right)}{R_{H}},$$ (25)

where

$$I_{\text{SD}}=\int_{y_{\min}}^{y_{\max}}J_{t}\left(y\right)\;dy=I\left(y_{\max}\right)+I\left(y_{\min}\right),$$ (26)

and

$$\Delta I=I\left(y_{2}\right)-I\left(y_{1}\right).$$ (27)

**Fig. 10:**
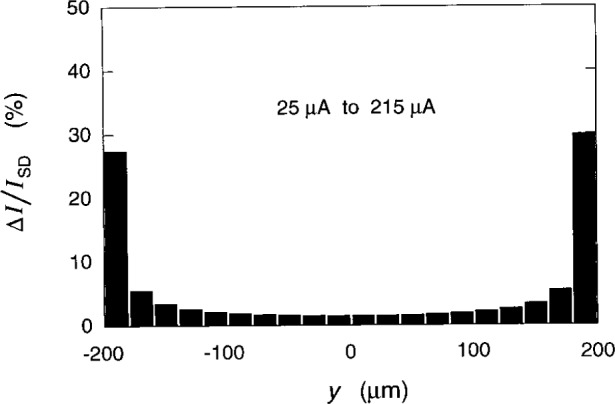
Percentages of the total current in twenty equally spaced segments across the device width for *I*_SD_ = 25 μA and 215 μA. The distributions are identical for the two currents.

Cage and Lavine [17] also determined the percentage of the total current in each of 20 equal segments across the 400 μm wide device. Their results for *I*_SD_ = 25 μA and 215 μA for the *i* = 2 plateau at 12.3 T are reproduced in Fig. 10. The current distributions are nearly symmetric across the device, and are virtually identical between 25 μA and 215 μA.

The edge-channel current could only be along the right-hand side segment in Fig. 10 for these current and magnetic flux density directions, but 70 % of the current is in the 19 segments to the *left* of this segment. Also, the edge-channel current would by necessity be within the confining potential regions, but 97 % of the applied current is in the region between − *λ* and *λ*, which is *outside* of the confining potentials. Therefore, a large fraction of the applied current is in the device interior. (This conclusion also follows directly from the experimental potential distributions of Fontein et al. [13], whose measurements are independent of any model assumptions.)

A plot for the current distribution at *I*_SD_ = 0 μA is not shown here, but there would be − 0.81 μA and + 0.81 μA in the left-hand and right-hand side segments, respectively, because *I*_c_(rhs) = − *I*_c_(lhs) = 0.81 μA. No current exists in the other eighteen segments for this case.

## 6. Discussion and Conclusions

The potential distribution across the width of a quantum Hall effect device has been modeled as a combination of confining potentials along the device sides and a charge-redistribution potential. The confining potential is due to surface charges and charge-depletion near the device edges. The charge-redistribution potential arises from the applied current. Normally, there would be insufficient information to uniquely define the total potential, but by using breakdown data at large applied currents [17, 20] an example was found where the potential could be obtained with no free parameters. The resulting potential distribution is in excellent agreement with experiment [13].

Using this potential distribution, I predict that skipping orbits do not occur in quantum Hall devices. Indeed, the nearest current-carrying occupied states are 55 to 60 cyclotron radii away from the physical device edges for currents smaller than the critical current for the onset of breakdown.

Edge-channel states are a useful mathematical construct, especially for very small applied currents. However, edge-state models only predict that the applied current is proportional to the difference in chemical potential across the device, not how the current is distributed across the device [29]. One must know the potential distribution to obtain the current distribution.

Can edge-channel states be distinguished from bulk states? Yes, if we define which states are at the “edges” and which are in the “interior” region. The edge states must be entirely within the confining potential regions because that is where the current is located when a device is placed in a magnetic field and there is no applied current. Therefore, let us assume that the “edge” states are composed of current-carrying occupied states of the two confining potentials on either side of the device and of the charge-redistribution potential, and that these “edge” states are in the two regions between *λ* and *y*_max_ and between − *λ* and *y*_min_. For the examples in Sec. 5.3 at 25 μA and 215 μA we find that only 4.9 % and 2.7 % of these two applied currents are located between *λ* and *y*_max_ and only − 1.9 % and 0.3 % are between − *λ* and *y*_min_. (The − 1.9 % value means that the current is in the − *x* direction over this region of the device at 25 μA.) Furthermore, 97 % of the applied current is in the region between − *λ* and *λ* where the edge-channel current cannot exist. Therefore, nearly *all* of the applied current is within the device interior, and not at the device edges. This conclusion is independent of our particular definition of the potential distribution since an identical current distribution can be obtained directly from the data of Fontein et al. [13].
